# Optic nerve and choroidal calcification in pediatric neurofibromatosis type 2

**DOI:** 10.1016/j.ajoc.2025.102306

**Published:** 2025-03-25

**Authors:** Shreya Gupta, Goura Chattannavar, Ashima Goyal Sharma, Ramesh Kekunnaya

**Affiliations:** a Jasti V Ramanamma Children's Eye Care Centre, Child Sight Institute, L V Prasad Eye Institute, Hyderabad, India; b Standard Chartered Academy of Eye Care Education, Clinical Fellow, L V Prasad Eye Institute, Hyderabad, India; c P C Sharma Eye Hospital, Ambala, Haryana, India

## Abstract

We describe a teenage boy with gradually progressive blurred vision in right eye. He could appreciate hand motions in the right eye and 20/20 vision in the left eye. Ophthalmic examination revealed bilateral optic atrophy with yellow-white peri-papillary lesion in the right eye. Computed Tomography (CT) brain and orbit showed choroidal, optic nerve and intracranial calcifications. The Magnetic resonance (MR) imaging showed the calcification selectively involved the optic nerve sheath with no abnormal hyperintensity or enhancement of the optic nerve substance, suggestive of a bilateral optic nerve sheath meningioma.

The work-up for metastatic calcification was normal. The molecular genetic testing revealed a heterozygous, missense variant in *NF2* (c.784C > G, p. Arg262Gly) gene, which segregated in the proband's mother. This variant is not reported in the literature and adds to the genotype of Neurofibromatosis Type 2. To preserve the vision in left eye, the child underwent external beam radiotherapy (EBRT). The vision and fields in the left eye are stable after EBRT at a two-year follow-up. This case highlights the systematic approach to a case of optic atrophy in a child and identifying the rare etiology of optic nerve calcification with a report of novel variant in *NF2* gene.

## 1 Introduction

Optic atrophy is essentially a morphological sequelae to a defect in the afferent visual pathway system by the virtue of retinal ganglion cell loss.^1^ Brodsky and colleagues have reported an exhaustive list of optic atrophy in children which includes congenital optic atrophy, compressive lesions, infiltrative lesions, hydrocephalus, craniosynostoses, GAPO syndrome, fibrous dysplasia, hereditary causes including dominant optic atrophy, Wolfram syndrome, mitochondrial causes of optic atrophy including Leber hereditary optic neuropathy, neurodegenerative disorders, secondary causes like hypoxia, ischemia following perinatal injury and traumatic optic neuropathy.^2^ Though there have been few Indian studies depicting the etiology of childhood blindness, specific causes of optic atrophy have not been addressed elaborately. Chinta S. et al. reported hypoxic ischemic encephalopathy (HIE) as the most frequent cause of childhood optic atrophy. The second most common cause of optic atrophy was idiopathic with normal neuroimaging. However, due to economical constraints and poor follow up, genetic work up and familial screening was not done in this study which may have underestimated the number of hereditary optic neuropathy cases. Other causes reported in this study included hydrocephalus, both primary and secondary to tumors and tubercular meningitis. Other infective causes included viral meningoencephalitis and TORCH infection. Less common causes reported in this study were post traumatic, craniofacial malformations, demyelinating optic neuritis, ophthalmic artery occlusion and blood malignancies.^3^

We describe a case of a teenage boy who presented with gradual progressive vision loss in right eye since early childhood. Further investigations revealed concurrent optic nerve and intracranial calcifications. We describe our approach and the change in diagnostic momentum with subsequent investigations and arriving at the final diagnosis.

## 2 Case details

**Fig. 1:**
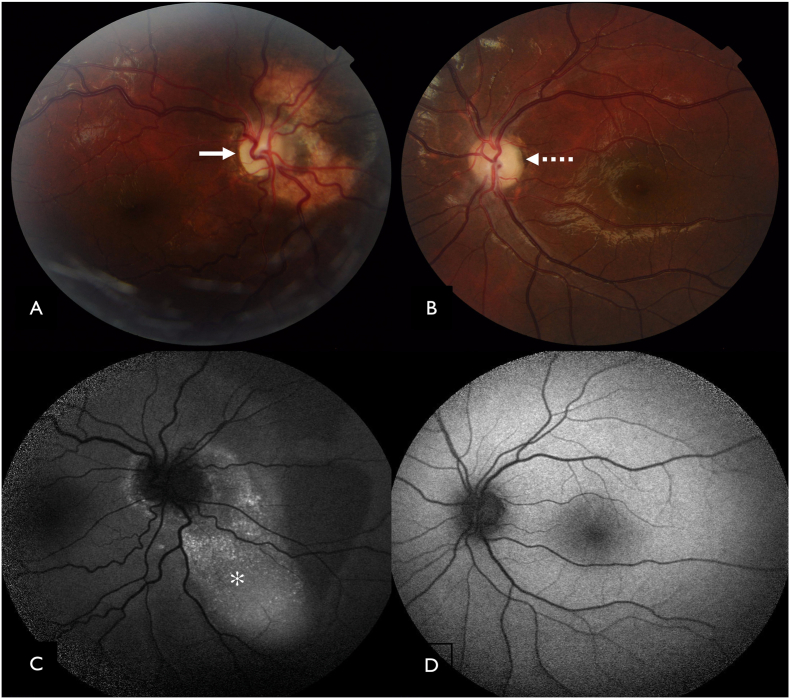
(A) Color fundus photograph of the right eye shows diffuse disc pallor with well-defined yellowish raised lesion in peripapillary area with overlying pigmentary changes (white arrow).(B) Color fundus photograph of the left eye reveals diffuse pallor of the optic disc (dashed arrow). (C) Fundus autofluroscence of the right eye shows, stippled hyper autofluorescence corresponding to the raised yellow lesion on color fundus photograph (asterix). (D) Fundus autofluorescence of the left eye shows normal pattern of autofluorescence. (For interpretation of the references to color in this figure legend, the reader is referred to the Web version of this article.)

A 14-year-old boy born to a non-consanguineous married couple presented with gradual progressive loss of vision in right eye since early childhood. His birth history was uneventful and attained developmental milestones at appropriate age. He could appreciate hand movements in the right eye and 20/20, N6 in the left eye. Color vision was normal in left eye and could not be tested in right eye due to poor vision. Orthoptic evaluation revealed 20PD (Prism diopter) esotropia on modified Krimsky test with full extraocular movements in both eyes. Pupil examination showed afferent pupillary defect in right eye. Anterior segment of both eyes had no signs of active or past inflammation. No lenticular opacities were noted in both eyes. Fundus examination of right eye (Fig. 1A) revealed medium sized optic disc with diffuse pallor and a well-defined yellowish raised lesion in the peripapillary area with overlying pigmentary changes and normal retinal vessels. Rest of the posterior pole and peripheral retina was normal. Left eye (Fig. 1B) fundus evaluation revealed diffuse pallor of the optic disc and the rest of retina was normal. A neurologic examination showed no focal motor and sensory deficit. He did not complaint of tinnitus, vertigo or balance issues. He had no hearing loss.

**Fig. 2:**
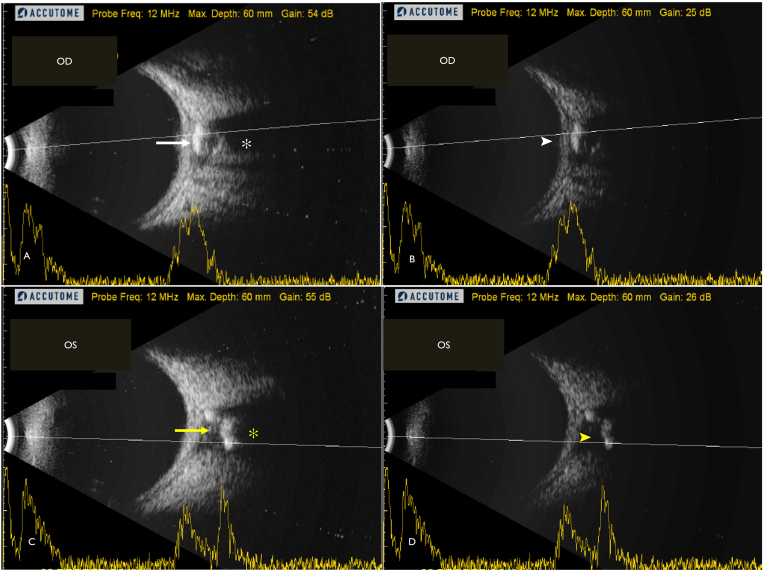
(A, C) Ultrasound-B scan of the right eye shows a hyperechoic lesion present posterior to optic nerve head and in the peripapillary area (solid white arrow) with posterior acoustic shadowing (white asterix) in the right eye (OD) and the left eye (OS) (solid yellow arrow and yellow asterix) respectively. (B, D) Ultrasound-B scan of the right eye (OD) and the left eye (OS) shows persistence of hyper echogenicity (white and yellow arrow-head) at adjusted low gain of the ultrasound B scan. (For interpretation of the references to color in this figure legend, the reader is referred to the Web version of this article.)

Fundus autofluorescence revealed stippled hyper autofluorescence corresponding to the yellowish lesion in right eye (Fig. 1C) and a normal pattern of autofluorescence in the left eye (Fig. 1D). With the suspicion of calcified nature of the lesion, an ultrasound-B scan was advised, which showed a hyperechoic lesion present posterior to optic nerve head and in the peripapillary area with posterior acoustic shadowing (Fig. 2A) which persisted even on low gain settings (Fig. 2B) indicating presence of a calcified choroidal lesion in the right eye. A similar hyper echoic lesion was seen posterior to optic nerve head in the left eye (Fig. 2C & D). However, the lesion was more proximal as compared to the right eye. This explains why the lesion was not seen on clinical fundus examination as well on autofluorescence images.

**Fig. 3:**
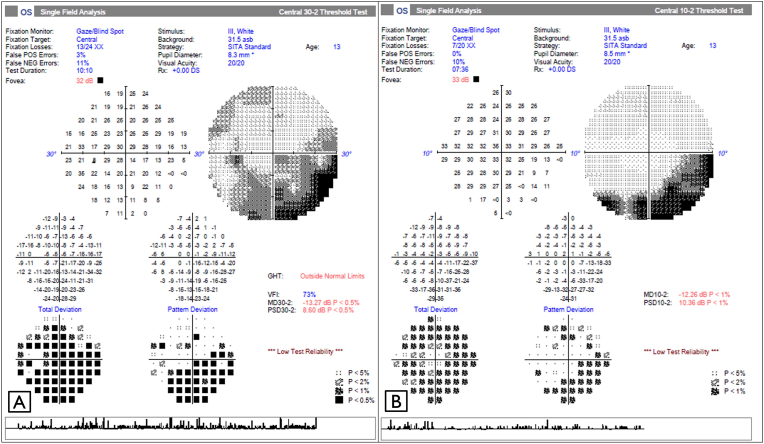
Humphrey visual field analysis of the left eye demonstrating (A) a nasal and inferior depressed points on 30-2 and (B) a corresponding inferior field defect on 10-2.

A Humphrey visual field analysis of the left eye demonstrated a nasal and inferior depressed points with a foveal threshold of 33db (decibel) (Fig. 3). Visual field analysis could not be obtained in the right eye due to poor vision.

**Fig. 4:**
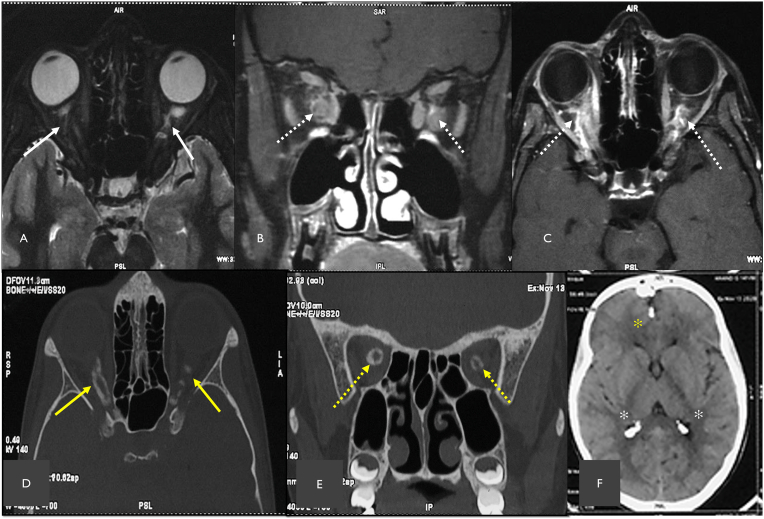
Magnetic resonance imaging of the orbit (A) T2-weighted mid-axial section shows an ill-defined lesion in the intra orbital segment of optic nerve on either side (solid white arrow); T1-weighted with gadolinium contrast of the mid orbit coronal section(B) and mid-axial section(C)shows thick and heterogenous enhancement of the optic nerve on both sides (dashed white arrows). (D) Computed tomography in the bone window of mid-axial section of orbits shows hyper-density along the optic nerve sheath of right and left optic nerve (yellow arrow); (E) shows ring shaped hyper-density encasing the right and left optic nerve (dashed yellow arrow), (F) Computed tomography of brain shows intracranial calcification of flax cerebri (yellow asterix) and choroid plexus of lateral ventricle (white asterix). (For interpretation of the references to color in this figure legend, the reader is referred to the Web version of this article.)

Considering the history, clinical signs and findings on the investigations, bilateral choroidal osteoma was our first differential diagnosis followed by metastatic calcification. The features not favoring choroidal osteoma were bilateral involvement, absence of choroidal neovascularization, and absence of serous retinal detachment and the lesions were proximal to the level of choroid in left eye on B scan. Magnetic resonance imaging (MRI) of both orbits showed an intra orbital ill-defined hyperintense lesion on both sides on T2 weighted sequence (Fig. 4A) which showed heterogenous enhancement on post gadolinium contrast T1 weighted sequences (Fig. 4B & C) suggestive of an optic nerve sheath meningioma. To delineate the extent of calcification and to look for other foci of calcification, computed tomography of brain and orbit was advised. Orbital sections seen in bone window revealed asymmetric ring like and “tram-track” like calcification in both eyes encasing the intra orbital part of optic nerve (Fig. 4D & C). Intra-cranial calcified lesions were seen in falx cerebri and choroid plexus (Fig. 4F)

With the multiple regions of intra-orbital and intra-cranial calcifications, he was investigated for metastatic calcification. Serum phosphate values were 5.6mg/dL which were borderline raised. Serum Vitamin D was 34.60ng/dL, Serum Calcium was 10.1 mg/dL, Serum Parathormone was 34.65 pg/dl, Serum Creatinine was 0.5 mg/dl and Blood urea Nitrogen was 9.8 mg/dL, all of which were in the normal range. With the suspicion of optic nerve sheath meningioma, whole exome sequencing (Sandor Specialty Diagnostics Pvt. Ltd, Hyderabad, India) on blood of the proband revealed a heterozygous, mis-sense variant of uncertain significance at exon 8 in *NF2:* c.784C > G (p. Arg262Gly) gene. Segregation analysis of parents revealed detection of same variant in *NF2* gene in mother, however she was asymptomatic.

Based on these findings, a diagnosis of bilateral optic nerve sheath meningioma with genetically confirmed NF2 was made. Based on the diagnosis of bilateral optic nerve sheath meningioma, external beam radiotherapy (EBRT) was instituted for both eyes He received 54Gy/30 fractions in right eye and 50.4Gy/28 fractions in the left eye followed by similar dose 3 months later. Precautions associated with one-eyed status was explained and polycarbonate glasses were prescribed for protection. His asymptomatic sibling was screened and had no clinical signs of Neurofibromatosis type 2. At follow-up of two years after EBRT, his vision in the right is status-quo and maintained 20/20, N6 in the left eye with stable visual fields.

## 3 Discussion

Pathological calcification refers to abnormal deposition of calcium salts in tissues. When these deposits occur in degenerated and necrotic tissues with normal calcium metabolism, it is called dystrophic calcification. In contrast, metastatic deposition occurs in normal tissues with deranged calcium and phosphorous metabolism, often due to end stage renal disease, hyperparathyroidism and hypervitaminosis D.^4^

A third type, metaplastic calcification, is specific to dural tissue wherein there is idiopathic osteoblastic metaplasia of dural mesothelial cells.^5^ Intracranial calcifications of brain parenchyma or vasculature are reported in 1 % of young individuals.^6^ Physiologic pediatric intracranial calcification is reported in choroid plexus, habenula, tentorium, falx cerebri and pineal gland.^7^^,^^8^ Calcified lesions along the optic nerve can occur in optic nerve sheath meningioma, optic nerve head drusen and idiopathic dural optic nerve sheath calcification with rarer causes including calcified optic nerve gliomas and metaplastic dural ossification.^9^^,^^10^

Lingappa et al. reported a rare case of *FGF23* associated autosomal recessive familial hyperphosphatemic tumoral calcinosis in an 8-year-old who had serial manifestations of facial paralysis, decreased vision in both eyes, fracture of right elbow, crystal deposits on the lid margins and interpalpebral conjunctiva, optic nerve sheath and intra-cranial calcification.^11^

Idiopathic duro-optic calcification is an entity described by Phadke et al. in 1996 which includes presence of optic nerve or optic nerve sheath calcification along with concurrent intracranial calcifications and progressive optic atrophy.^10^ The label idiopathic at that time can be attributed to lack of good quality imaging which may have led to underdiagnosis. The two cases reported by them underwent extensive work up for metastatic calcification as well, revealing no gross abnormality^12^

Whole exome sequencing in our patient revealed a heterozygous missense variant at exon 8 of *NF2* gene. Although the variant in *NF2* originally reported as a variant of uncertain significance, on further analysis was a likely pathogenic variant. The *NF2*: c.784C > G (p. Arg262Gly) is not reported in population databases of gnomAD, TOPMed Bravo, GME Variome, 1000 Genomes, GenomeAsia and India DB. Pathogenic variants in this gene are associated with NF 2 which is characterized by nervous system and skin tumors and ocular abnormalities. The current variant is a novel variant with no clinical reports in literature. The variant is conserved across all species. The variant is reported deleterious by various insilico models (Revel, Varity, DANN, MetaLR, PrimateAI, BayesDel MT Med: MUT Assesor, GenoCanyon, fitCons). Other nucleotide changes at the same codon are reported in ClinVar database by multiple providers with no conflicts as pathogenic in c.784C > T (p. Arg262Ter) causing NF2, uncertain significance of c.785G > A (p.Arg262Gln) in *NF2* and a single provider on c.786A > G (p.Arg262 = ) as likely benign. The segregation in the mother, absence of any reports in population database, deleterious variant on insilico prediction tools and high conservation across species resulted in reclassifying the variant as likely pathogenic. The variable penetrance in autosomal dominant inheritance explains the mother being asymptomatic. The current variant adds to the genotype of Neurofibromatosis type 2. 50–75% of patients with NF2 develop meningiomas.^12^ The *NF2* variant in the current report may have a predilection for optic nerve sheath meningioma, retinal and choroidal hamartomas that predispose to calcification which may disguise as choroidal osteoma, and metastatic calcification.

Husum et al. reported a case of a 48-year-old male who was diagnosed as idiopathic duro-optic calcification, but serial neuroimaging confirmed the diagnosis of optic nerve sheath meningioma but lacked the genotyping of the case.^13^. Boltshauser et al. reported presence of morning glory disc anomaly in 2 cases of suspected NF2. They reported presence of intracranial schwannomas, vascular hamartomas at disc and peripapillary arteriovenous communications as well. However, these cases were not gene determined cases.^14^ The final diagnosis of optic nerve sheath meningioma in our patient mandated irradiation therapy. Our case not only highlights the rare occurrence of optic nerve and choroidal calcification in *NF2* but also reports the novel likely pathogenic variant in *NF2* which guided the management.

## 4 Conclusion

• Optic atrophy accompanied by optic nerve and choroidal calcification may be a presenting feature of NF2.
• Hereditary and metastatic causes of optic nerve calcification should be investigated.
• Timely diagnosis of optic nerve sheath meningioma in NF2 and appropriate referral for EBRT is necessary to save the vision.

## Declaration of figures’ authenticity

All figures submitted have been created by the authors who confirm that the images are original with no duplication and have not been previously published in whole or in part.

## CRediT authorship contribution statement

**Shreya Gupta:** Writing – review & editing, Writing – original draft, Validation, Supervision, Data curation. **Goura Chattannavar:** Writing – review & editing, Validation, Methodology, Data curation. **Ashima Goyal Sharma:** Writing – review & editing, Validation, Data curation. **Ramesh Kekunnaya:** Writing – review & editing, Validation, Supervision, Conceptualization.

## Patient perspective

Translated from Telugu language to English.

‘I had very poor vision in right eye for which I visited LV Prasad eye institute, Hyderabad campus. They investigated in detail, and I was told there was a benign tumor in my right optic nerve. As advised, I went ahead with radiation therapy. Soon after, I could appreciate some visual improvement and was able to recognize faces from my right eye. They started radiation therapy in both eyes to preserve vision in my left eye. I am happy that timely treatment helped in saving the vision in my left eye.’

## Patient consent

Consent to publish this care report has been obtained from the patient in writing in accordance with the Elsevier patient consent policy.

## Institutional review board

An ethical clearance was obtained from the Institutional review board for the current report.

## Acknowledgements and disclosures

The authors would like to thank Dr Brijesh Takkar and Dr Swathi Kaliki for co-managing the patient.

## Authorship

All authors attest that they meet the 4 current ICMJE criteria for Authorship.

## Funding

The Hyderabad Eye Research Foundation.

## Declaration of competing interest

The authors declare that they have no known competing financial interests or personal relationships that could have appeared to influence the work reported in this paper.
